# Connector-Free World-to-Chip Interconnection for Microfluidic Devices

**DOI:** 10.3390/mi10030166

**Published:** 2019-02-27

**Authors:** In-Hyouk Song, Taehyun Park

**Affiliations:** 1 Department of Engineering Technology, Texas State University, San Marcos, TX 78666, USA; in-hyouk.song@txstate.edu; 2 School of Mechanical Engineering, Kyungnam University, Changwon 51767, Korea

**Keywords:** world-to-chip interconnection, connector-free, microfluidic, interference fit

## Abstract

In the development of functional lab-on-a-chip (LOC), there is a need to produce a reliable and high pressure connection between capillary tubes and microfluidic devices for carrying fluids. The current technologies still have limitations in achieving ideal interconnection since they are bulky, expensive or complicated. In this paper, a novel connector-free technique using an interference fit mechanism is introduced for world-to-chip interconnection. The proposed technique has considerable potential for replacing current interconnection tools for microfluidic devices due to the advantages including no chemical contamination, easy plugging, enough strength to sustain pressure, high density integration, simple and rapid integration.

## 1. Introduction

In the last decade, a large number of scientists have been involved in the development of microfluidic technology, also called lab-on-a-chip (LOC). Due to the massive efforts, in recent years the miniaturization of existing biological research systems and tools has facilitated the progress of biology [1]. Microfluidic technology plays an essential roles in analyzing biological systems. Since the concept of a miniaturized total chemical analysis system was introduced [2], advanced miniaturization techniques have been proposed and demonstrated in a variety of innovative approaches to integrate multiple functions of systems on a common substrate [3]. Due to the advantages such as small reagent consumption, disposability, short reaction time, and portability, the multifunctional microchip has been researched for diagnostics, analytical chemistry, biohazard detection, genomic research, and environmental monitoring applications using electrical, mechanical, or optical detection technologies [3,4,5]. To complete the development of a functional LOC device, one of the crucial processes is a reliable world-to-chip interconnection between the microchips and peripheral devices through capillary tubes [6]. Over the past decades, there have been numerous efforts to develop the proper microfluidic interconnection techniques, which are categorized into irreversible [7,8,9,10] and reversible [11,12,13] methods. While the former has been employed to permanently attach the individual microfluidic system to the external fluidic connector tubes, the latter enables engaging and separating the world-to-chip interface. 

Considerations for ideal fluidic interconnection are minimal dead volume, easiness of plugging/unplugging, reliability at high pressures, simple and low-cost techniques, and compatibility with commercial tubes and fittings [6]. However, current technologies still have limitations in achieving this ideal interconnection. For example, the commercially available irreversible connectors, for example, Nanoport^TM^ (IDEX Corp., Lake Forest, IL, USA), are bulky, expensive and cumbersome. The commercial Nanoport^TM^ connector is required to be bonded on a microfluidic substrate and it occupies an area of at least 55.4 mm^2^. Large connectors are correlated with large dead volumes, even though the connection can withstand high pressure [14,15]. The use of glue or chemical to bond connectors is another disadvantage for chemical/biological analysis applications in terms of a potential chemical contamination. An epoxy-free process was reported to form a connector between capillary tube and microfluidic motherboard with flanging operation. It, however, requires an additional heating process to form the connector [16]. Alternative techniques emerged to achieve reliable and reversible macro-to-micro interfaces: polydimethyl siloxane (PDMS) based chips, using needles or capillaries directly with microholes of PDMS devices [8,17,18,19,20,21,22]. The connecting mechanism using a needle has advantages of easiness of plugging, reusability, high density connection and simple and low-cost connection. However, the interconnections suffer from low leakage pressures unless very thick layers are used [17]. 

In this paper, we improved a connector-free interconnection technique for a microfluidic device using an interference fit mechanism, which is a fastening between two parts in which the external dimension of the inserting part slightly exceeds the internal dimensions of the hole. Since the larger inserting part is forced into the hole of smaller dimensions, both parts are forced to fit together and extremely high friction and pressure results between them. The friction allows the interconnection to sustain high pressure. The interconnection does not require the use of glue or the assembly of an external connector, but the microtube is directly integrated into the microfluidic chips. Use of the interference fit has already been reported for connecting capillaries to microfluidic devices [23,24,25]. However, the proposed interconnection technique in this paper achieves a simple fabrication process, the easy plugging and removing tube, and low dead volume, but enough strength to sustain high pressure.

## 2. Design Concept 

**Figure 1:**
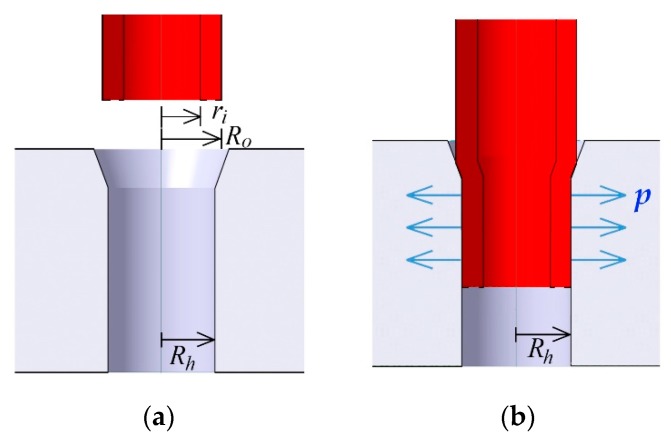
The conceptual illustration using an interference pressure connection. (**a**) before and (**b**) after interconnection. *R_o_* is the outer radius of the capillary, *R_h_* is the radius of the microhole, and *r_i_* is the inner radius of the inserted capillary. *p* indicates the interference pressure.

Figure 1 illustrates the process scheme of the proposed interconnection technique for integrating microcapillaries into the microhole of the microfluidic chip. For an interference fit, the outer radius of the capillary, *R_o_*, is larger than the radius of the microhole, *R_h_*, by the radial interference, (*R_o_* − *R_h_*). An oversized capillary is inserted into a microhole, and the capillary deforms due to the squeeze of the microhole. However, the inlet of the microhole must be formed larger than the outer diameter of the capillary to let the capillary in without bending. Hence, a countersink shape is chosen to make a larger inlet for the microhole, as shown in Figure 1a. The tapered inlet helps to guide the capillary into the microhole without bending, and the smaller diameter hole seals the plugged-in capillary with interference pressure.

The rigidity of the microholes is expected to be greater than that of the capillary, so mechanical deformation occurs primarily at the capillary. The capillary is inserted into the hole of microchips by shrinking or press fitting. After insertion, an interference pressure develops between the capillary and the microhole at the nominal radius *R_h_*, causing radial stresses, *p*. This pressure is given by [26]:

(1) $$p=\frac{R_{o}-R_{h}}{R_{h}\left[\frac{1}{E_{m}}\left(1+v_{m}\right)+\frac{1}{E_{c}}\left(\frac{R_{h}^{2}+r_{i}^{2}}{R_{h}^{2}-r_{i}^{2}}-v_{c}\right)\right]}$$

where *E* and *v* are Young’s modulus and the Poisson’s ratio of the materials, respectively. The subscripts of *m* and *c* stand for microfluidic chip and capillary, respectively. As shown in Figure 1a, *r_i_* is the inner radius of the capillary before insertion. The capillary tube is plugged manually to the microchips. The countersink approach offers an easy-to-integrate and applicable microfluidic connection with the plug-and-play feature. Here, we have tested four different tapered tip angles and measured pull-out force, which informs the strength of connection. 

**Figure 2:**
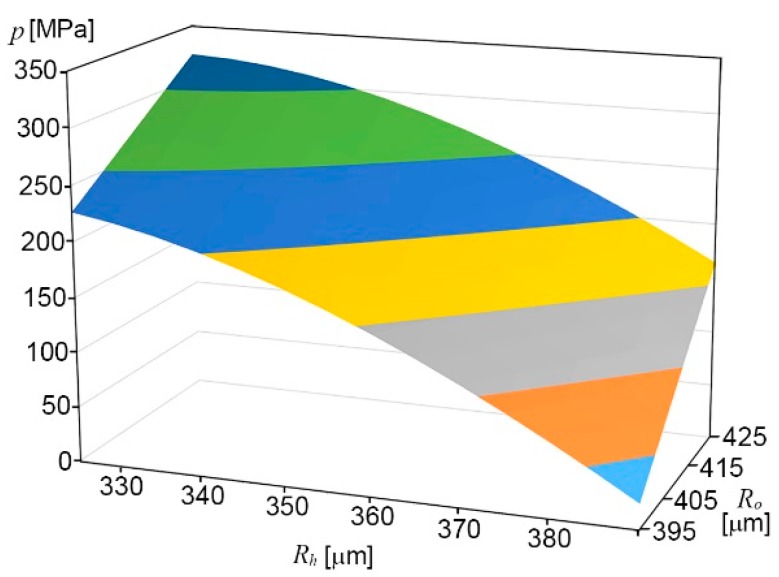
Interference pressure, *p*, as a function of *R_h_* of P polymethyl methacrylate (PMMA)-microhole, and *R_o_* of PEEK-capillary, for *r_i_* = 250 µm.

In this research, the PEEK capillary (*E_c_* = 3.7 GPa, *v_c_* = 0.3779) was chosen, which is one of the commonly used materials in microfluidics research, along with PTFE and Teflon [9,12,27,28,29,30,31]. The capillary tube was inserted into a microhole in a polymethyl methacrylate (PMMA) substrate (*E_m_* = 3.2 GPa, *v_m_* = 0.37). For example, the inference pressure is analytically 153 MPa for the values of *R_o_* = 397 µm, *R_h_* = 360 µm, and *r_i_* = 250 µm, which are the actual dimensions we used in this experiment. Figure 2 shows a simulation result using Equation (1). The interference pressure is gradually decreased as the radius of the outer capillary is close to that of the microhole. The larger *R_o_* for a given microhole size, the higher pressure the interconnection shows.

## 3. Results and Discussion

### 3.1. Interconnection

Thermoplastic polymers feature a variety of material properties for meeting the demands of microfluidics devices. Moreover, the fabrication techniques for polymer microfluidic devices, such as hot embossing and injection molding, are relatively simple and do not require hazardous etching reagent compared to glass substrate or silicon substrate. Hence, a variety of thermoplastic polymers have been used in research on bio/chemical applications using microfluidic devices. In order to demonstrate the proposed interconnection technique, PMMA is employed, which has become one of the dominant materials for fabrication of microfluidic devices, primarily due to its low cost and excellent optical and mechanical properties [32,33].

**Figure 3:**
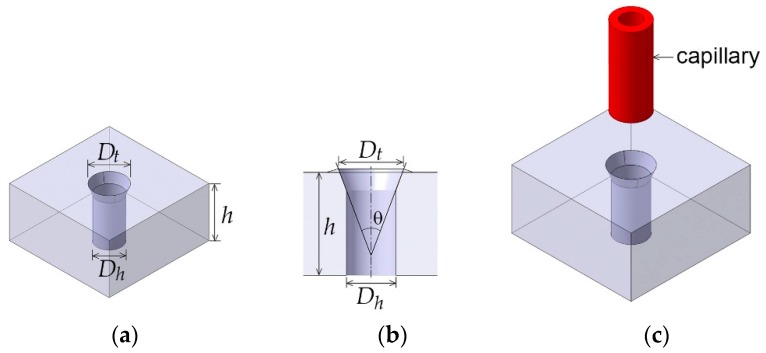
(**a**) Drilled microhole, (**b**) countersink microhole, and (**c**) inserting capillary into the countersink microhole. Here, *D_t_* is diameter of tapered top and *D_h_* is diameter of microhole. θ is the tapering angle. *h* is the thickness of the substrate.

Figure 3a,b show a 3D image and a cross-section view of the countersink type microhole, respectively. Here, *D_t_* is the diameter of tapered top and *D_h_* is the diameter of microhole. θ indicates the tapering angle. The thickness of substrate is *h*. Figure 3c illustrates the insertion of capillary into the microhole. For this experiment, a 1.65 mm thick PMMA was used for a substrate of the microfluidic chip, and PEEK Tub (#1569, IDEX Corp., Lake Forest, IL, USA) was prepared for a capillary whose outer diameter (OD) and inner diameter (ID) are 794 µm and 500 µm, respectively. The thickness of the capillary is 147 µm. To fulfill the interference fit, the diameter of the drilled hole should be smaller than the OD of the inserting capillary. A 0.71 mm diameter drill bit was chosen to create microholes in the PMMA substrates, resulting in *D_h_* of around 720 µm. The diameter of the microhole can be scalable unless the diameter is larger than the OD of the capillary and smaller than double the capillary thickness. 

**Figure 4:**
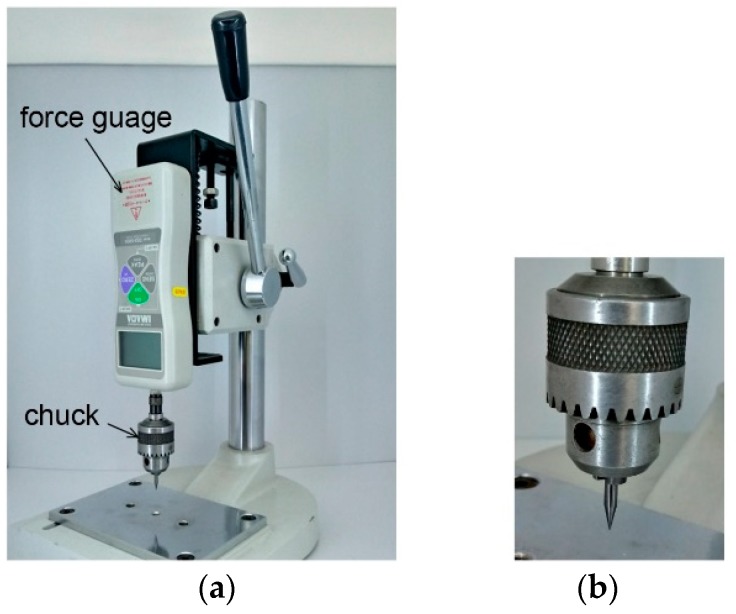
(**a**) Push-pull gauge stand assembled with a force gauge. A chuck is mounted on a spindle. (**b**) Inserted tapered tip into the chuck.

In order to form a countersink microhole, the cone-shaped bit was inserted into the chuck and tightened, as shown in Figure 4. The integrated chuck was attached to a force gauge, DS2-500N (IMADA Inc., Northbrook, IL, USA), mounted on a push-pull gauge stand to monitor the applied tapering force. The cone-shaped bits were aligned with the microholes of the microfluidic chips and pressed to form a tapered inlet with four different angles of 23.2°, 31.9°, 35.2°, and 41.8°. Figure 4b is the close-up view of the inserted cone bit of 23.2°. According to the experiment, more than 130 N of tapering force was required to form a larger diameter of tapered top than that of the capillary tube. Hence, the tapering force of the cone bit is increased slowly and released at 150 N to make a larger tapered top. The diameter of the tapered top, *D_t_*, is measured using Nikon MM-400 measuring microscope. The values measured are described in Table 1. 

**Figure 5:**
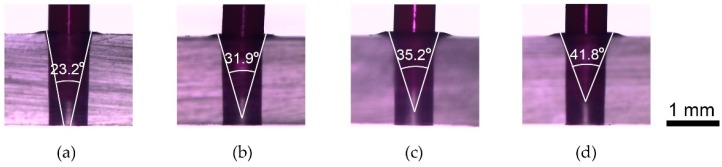
The side-view of capillary inserted in microhole. (**a**) for 23.2° (**b**) for 31.9° (**c**) for 35.2° (**d**) for 41.8°.

The following step is insertion of the capillary into the countersink microhole. The capillary could be directly inserted by hand. However, to compare with the effect of tapered angles for interconnection strength, a constant inserting force was necessary. For the insertion of the capillary, the integrated cone bit of Figure 4 was replaced with a PEEK Tube. Interference fit requires an adequate force to insert the capillary tube into the microhole. To determine the magnitude of this insertion force, the capillary was placed in the chuck instead of the tapered tip of Figure 4b. Then, the capillary was grabbed by the fingertips and pulled down vertically until there was no longer an increase of force at 13 N, which was chosen as an insertion force. Figure 5 is the side-views of the capillaries being inserted in the microholes for the tapered tip angles of 23.2°, 31.9°, 35.2°, and 41.8°, respectively. Experimental trials were conducted with three repetitions, and the measured diameters, tapering force and insertion force for each angled countersink inlet were recorded in Table 1. 

### 3.2. Interconnection Strength

**Figure 6:**
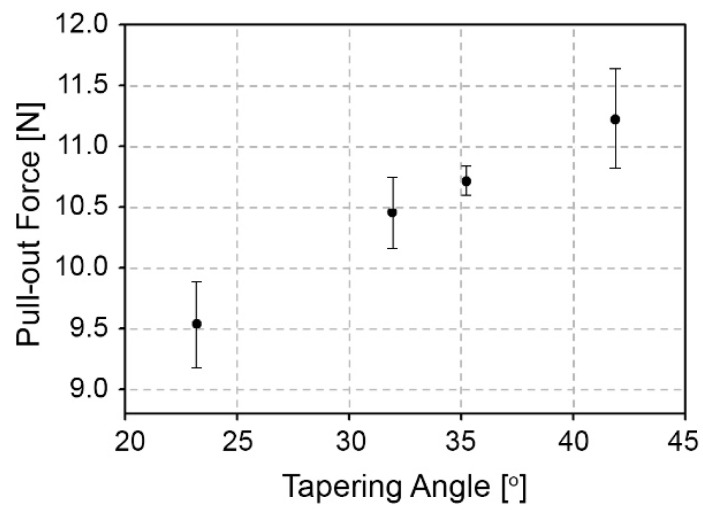
The measured pull-out forces are 9.54 ± 0.38 N, 10.45 ± 0.27 N, 10.72 ± 0.12 N, and 11.23 ± 0.44 N for tapering angles of 23.2°, 31.9°, 35.2°, and 41.8°, respectively.

In order to evaluate the interconnection strength of the proposed method, a pull-out force and a burst pressure tests were performed. The pull-out force was measured using a universal testing machine (DR-100, DR-TECH, Seongnam, Korea). For each specimen, the PMMA substrate was fixed and the capillary was pulled out at a speed of 2.5 mm/min until failure started. The pull-out force was measured from the maximum force. Figure 6 shows pull-out forces for tested angles with standard deviations. The pull-out forces were 9.54 ± 0.38 N, 10.45 ± 0.27 N, 10.72 ± 0.12 N, and 11.23 ± 0.44 N for tapered tip angles of 23.2°, 31.9°, 35.2°, and 41.8°, respectively. These pull-out forces are much higher compared to the previously reported PDMS-based press fitting technology [12,17], even though the chip thickness is much thinner. According to the results shown in Figure 6, the strengths of the connection were increased as the tapering angle increased. For the larger tapering angle, the capillary tube is inserted deeper under same insertion force of 13 N. This increases the contact area between the capillary tube and the sidewall of the microhole, resulting in the escalation of the pull-out force or the strength of connection.

The burst pressure of connection was also measured to evaluate the proposed interconnection strength. For the test, microfluidic channels were replicated using hot embossing technique on the pre-annealed PMMA sheet at 80 °C in an oven. The PMMA sheet and mold insert were placed onto the platen of the hot press (QM900M, QMESYS, Gyeonggi-do, Korea), configured to maintain 160 °C. They were fastened with a pressure of 8 MPa for 8 min. Following the separation of the PMMA chips from the mold insert at 80 °C, the chips were drilled to make microholes for interconnection with the capillary. After forming microfluidic channels, PMMA microchips were enclosed with a 250 µm thick polycarbonate (PC) film using an adhesive bonding technique [34]. For the adhesive layer, 7.5 g of PMMA beads (molecular weight: 75000, polysciences, Inc., Warrington, PA, USA) is dissolved in 300 g of propylene glycol monomethyl ether acetate (PGMEA). Then, PMMA solution is spin-coated on a PC sheet. Right after taking out the PMMA-coated PC sheet, the hot embossed PMMA chip is faced down on the PC sheet and pressed using a hand roller at room temperature.

Prior to the burst test, a leakage test was performed to make sure that the microfluidic device was leak-free, including interconnections. The capillary of inlet was connected to a syringe and air was blown while the device and the capillary of outlet were submerged in water. No leakage was observed at the interconnection regions of the inlet and outlet of the chip, and the bubbles flowed out at the end of the capillary of outlet. Then, the capillary of outlet submerged in water was sealed and the external capillary connected to compressed N_2_ gas through pressure regulators (Harris N_2_ gas regulator: 801, Cleveland, OH, USA). The regulator can support pressure up to 170 psi (= 1.172 MPa). The pressure of the compressed N_2_ gas was applied into the device channel through connector-free interconnection. By adjusting the regulator connected to the compressed N_2_ gas, gas pressure in the channel and the capillary was increased. Since the device was submerged in water, the bubbles came out if there was any leak or burst. Pressure was held at 170 psi for 20 min and released. The experiment was repeated several times. However, there were no bubbles observed and no failure occurred during the test. The pressure applied meant the inserted capillary expanded, and it enabled the connection to seal tightly and to hold such high pressure. 

**Figure 7:**
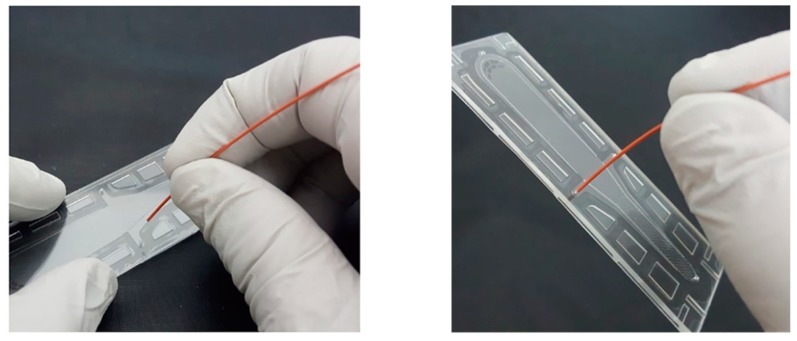
Connection procedure by hands. It takes less than 2 s.

**Figure 8:**
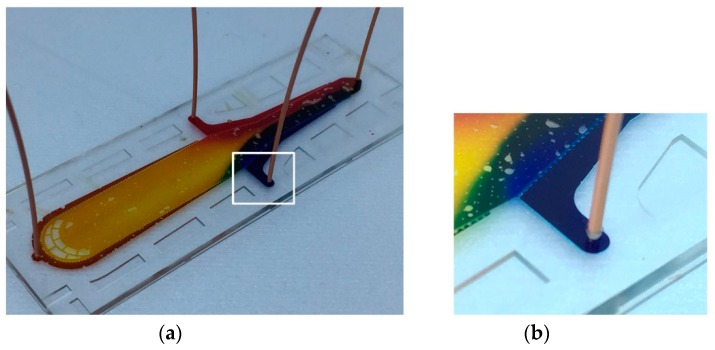
The microfluidic device connected with capillaries using the proposed connector-free interconnection technique and a close-up view of one of the interconnection.

Figure 7 shows how simple and how fast the interconnection is by hands. It took less than 2 s. The force of manual insertion is usually less than 13 N. The connection could withstand pressures in excess of 170 psi. The inserted capillary was then removed and inspected. The microholes did not show any visible damage. The capillary tube, however, was deformed at the inserted part, which was forced into the microhole. For the reversible test, the capillary was re-inserted into the microhole and measured by burst pressure test and leakage test. The device still functioned without any failure at 170 psi for 20 min. Finally, a microfluidic device connected with capillaries by the proposed connector-free interconnection was tested to demonstrate a real microfluidic application with three inlets and one outlet using three food dyes (blue, yellow, and red), shown in Figure 8.

## 4. Conclusions

For lab-on-a chip applications, there is a need to produce a reliable and high pressure connection between tubes and microfluidic devices for carrying fluids. This communication reports a connector-free interconnection technology applicable for microfluidic devices using the principle of interference fit as a seal between capillary and microfluidic chip. Compared to PDMS-based interconnection, the proposed connection technique needs a tapering process at the inlet of the microhole. However, the tapered inlet allows an easy plug-in with a strong connection. The capillaries can be integrated seamlessly into the microholes connected to the microfluidic channels. Integration of the capillary to microchip is straightforward and rapid. This technique can be extended beyond thermoplastic polymers and can be implemented on a silicon or glass substrate. Due to connector-free and direct integration, this approach can make a minimum dead volume, free from chemical contamination. The easy plugging and easy removing tube, with enough strength to sustain high pressures, is advantageous for low-cost, disposable microfluidic devices. Hence, the proposed technique would help researchers and scientists who work in microfluidic systems or BioMEMS. In this experiment, we demonstrated the novel connector-free technique with PEEK Tub (#1569, IDEX Corp., Lake Forest, IL, USA) only. However, a variety of materials and sizes are manufactured and used in microfluidics research as a capillary. In this reason, the proposed technique is necessary to fully explore and characterize interconnection strength tests on various materials and sizes in the future.

## Figures and Tables

**Table 1:** Experimental conditions and measured diameters of drilled holes and tapered tops.

Tapered Tip Angle (°)	*D_h_* (µm)	Tapering Force (N)	*D_t_* (µm)	Insertion Force (N)
23.2	722 ± 0.47	151.7 ± 0.47	952 ± 7.8	13
31.9	721 ± 1.89	152.3 ± 0.47	974 ± 1.9	13
35.2	720 ± 2.16	150.7 ± 0.47	1039 ± 11.5	13
41.8	721 ± 0.47	150	1049 ± 4.9	13
